# Exploring Spatial Variations in the Relationships between Landscape Functions and Human Activities in Suburban Rural Communities: A Case Study in Jiangning District, China

**DOI:** 10.3390/ijerph18189782

**Published:** 2021-09-17

**Authors:** Jie Zheng, Guodong Chen, Tiantian Zhang, Mingjing Ding, Binglin Liu, Hao Wang

**Affiliations:** 1 School of Landscape Architecture, Nanjing Forestry University, Nanjing 210037, China; zj_1229@njfu.edu.cn (J.Z.); chen35@njfu.edu.cn (G.C.); mjding@njfu.edu.cn (M.D.); 2 School of Architecture, Soochow University, Suzhou 215006, China; tiantzhang@suda.edu.cn; 3 The Collaborative Innovation Center of South China Sea Studies, Nanjing University, Nanjing 210037, China; DG1827018@smail.nju.edu.cn; 4 School of Geographic & Oceanographic Sciences, Nanjing University, Nanjing 210037, China

**Keywords:** suburban rural community, landscape function, human activities, GWR, OLS, ecosystem services

## Abstract

There is a complicated and contradictory relationship between landscape functions and human activities, especially in the suburban rural communities of metropolises. Previous studies focused on human interference to landscape function, ignoring the impact of landscape functions on human activities. Hence, the present study is focused on the impact of landscape function (based on ecosystem services) on human activities in suburban rural communities of China. The study evaluated the intensity of human activities based on big data; furthermore, the authors analyzed the spatial distribution characteristics through spatial autocorrelation, and probed into the spatial variations in the relationship between human activities and landscape functions using ordinary least squares (OLS) and geographically weighted regression (GWR) models. The result indicates that there are obvious spatial distribution differences in the intensity of human activities in suburban rural communities; that is, the intensity decreases from the inner to the outer suburban areas. Positive influencing factors of human activities are construction area, bus station, road network density, and leisure entertainment, among which, construction area is the principal driver; cultural heritage, hydrological regulation, and provision of aesthetics are negatively or positively correlated with human activities in various regions. The results offer insights for the sustainable development of rural environment in suburban areas and the big data-driven rural research.

## 1. Introduction

Due to the urbanization and rapid economic development, human needs and activities are increasingly diverse, which causes landscape to be increasingly multi-functional under the limited conditions of the natural ecosystem. As interfaces between urban settlements and rural hinterlands, the metropolitan suburban rural communities are characterized by complex landscape patterns and stronger landscape functional requirements [1,2], due to their impact on the economic, environmental, and social dynamics of cities [3]; for this reason, they often serve as arenas of conflicts of interest [4]. It is worth emphasizing that the type and quantity of landscape function (LF) vary depending on the type of landscape in suburban rural communities; hence, different management is needed [5,6]. Moreover, different LFs also have a huge impact on human activities. Therefore, for the successful landscape management and sustainable development of suburban rural communities, it is necessary to learn the relationship between human activities and the suburban rural LF for the purpose of studying the key factors of LFs affecting human activities.

LF is ecosystem service produced by spatial dominance and human behavior on a landscape scale. The LF is beneficial to human well-being [7,8,9,10], and is essential for the sustainable development of the human environmental system and natural resources. At present, “landscape service” and “LF” are normally considered to be “ecosystem service”; both terms can be substituted for each other in most cases [11,12]. “Ecosystem service” refers to the various benefits that human beings get from the ecosystem [13]. “LF” and “landscape service function” have different names but the same meaning. LF, however, can better describe the spatial composite system where nature and humans interact with each other, while ecosystem service focuses on natural systems. LF has been integrated into the EU’s decision-making process and spatial planning because it highlights the multi-dimensional characteristics of LF. Moreover, “landscape” is more attractive to the studies of non-ecological disciplines than “ecosystem service” [14]. In particular, suburban rural areas where human activities are intensive are no longer dominated by large areas covered by natural land, while ecosystem service can no longer replace LF; therefore, LF is adopted here instead of ecosystem service, and other factors such as cultural transportation are considered to be incorporated into the LF. LF in this study are described as processes and services that can provide direct or indirect benefits to human well-being. These are not provided by a particular ecosystem or landscape patch, but the result of interactions between different ecosystems and landscape structures within the landscape and between landscape environment and human activities. Lots of experts proposed the combination of the ecosystem and the physical and ecological characteristics of landscape, which led to an extensively used framework for evaluation [15,16,17]; however, they have not reached any clear consensus on finality and type.

Human activities can be defined as a series of behaviors that human beings take to the natural environment in order to meet the needs of their own survival and development, including development, destruction, protection and other activities [18]. Human activity has always been a general term that is difficult to quantify [19]. This is reflected in the challenges faced by human factors in the selection of indicators. Although some scholars evaluated population density [20], agriculture [21], etc. as substitutes for human activities, and tried to map the spatial pattern of human activities, such factors do not accurately and directly reflect human activities; capturing real data on human activities remains a challenge. Especially in China, where population density data are on a sub-district scale, it is difficult to acquire data on the scale of rural communities. The development of mobile Internet technology and big data provides opportunities for studies. For instance, mobile phone data [22], global positioning data (GPS) [23] and social media data [24] contain a lot of semantic information about human activities; in particular, mobile phone data helps depict the overall invisible spatial landscape [25]. Baidu Map, Amap, Tencent’s Easygo, and many other Internet-based map service platforms offer accurate human travel data, which are characterized by high permeability, wide spatial range and abundant human activity information (time and place) when compared with field survey data set [26]. It is worth mentioning that China has 989 million mobile phone users out of a total population of nearly 1.4 billion. The data of Internet platforms such as Baidu Heat map, Amap, and Tencent’s Easygo are all based on the traffic generated by mobile phone users, so it is called high permeability. For instance, the density of mobile phone users was used as a representative of human activities to study the impact of urban morphology on the vitality of Amsterdam. Li et al. used nighttime lighting data to show human activity space [27]. Baidu Heat Map has also been applied to many studies on Chinese cities [28,29].

The relationship between humans and LFs is closely integrated and complex. On the one hand, the demand for and supply of LFs are affected by humans [30], and human activities may change the ecological service system, thereby affecting the supply capacity of landscape service function [31], economic, cultural, and other factors of human desire [32,33]. The interaction between LF and humans has been increasing, and most studies are focused on the interference of human activities to the ecosystem service system and LFs [34,35,36] or between human disturbance and landscape pattern [37]. They seldom pay attention to the impact of LFs on human activities and the relationship between them; moreover, the spatial heterogeneity of LF is largely ignored. Without reasonable attention, the relationship between LFs and human activities is likely to be distorted [38]. Furthermore, it is not clear whether the relationship between human activities and the environment is linear or non-linear [39]. Thus, there is an urgent need to learn the interactions between them. For the analysis of the relationship between LF and human activities, most studies lack in-depth exploration of the driving factors. Big data offer unprecedented opportunities for studies on cities [40]; however, due to the confidentiality of data, most studies on rural areas are qualitative or semi-quantitative ones.

In this study, we propose a new framework to explore the relationship between LF and human activities. Taking the rural communities in Jiangning District, Nanjing as an example, the present study employs a type of big data (Baidu Heat Map) as the basis to further quantify the spatial characteristics of human activities from the perspective of rural community unit. Then, the authors analyzed the relationship between LFs and human activities on that basis, and explored the key factors driving human activities through geographic weighted regression. The present study is focused on the following topics: (1) The spatial distribution of human activities in suburban rural communities and its characteristics; (2) Exploration of the driving factors of the impact of LF on human activities.

## 2. Materials and Methods

### 2.1. Study Area

**Figure 1:**
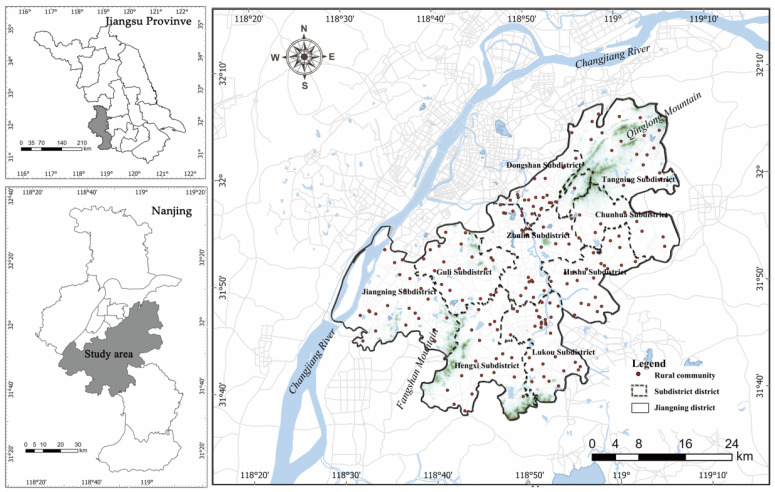
The location of the study area.

The study area is located in the southeast of Nanjing (Figure 1); as one of China’s megacities, Nanjing is not only a central city in eastern China, but is also a science and education center of China. The rapid urbanization of Najing raised the urbanization rate up to 83.2%, reaching a population of over 8 million. Nanjing has jurisdiction over 11 districts; the study area surrounds the main urban area of Nanjing from east, west, and south. It is a transportation hub for Nanjing’s external communication and is only 15 km away from Xinjiekou, a landmark at the city center. With a permanent resident population of 1.3473 million, Jiangning District has a jurisdiction over 10 subdistricts and 190 rural communities, covering a total area of 1561 square kilometers, which accounts for 24% of the total area of Nanjing. Jiangning District is the only typical case selected for the rural revitalization strategy in Jiangsu Province. It comprises 1500 natural villages; there are 73 city-level or higher-level cultural heritage protection areas in the study area, and the cultural heritage villages have greatly promoted the development of tourism in the area (33.06 million tourists received in 2017).

Jiangning District is rich in natural resources and diverse topographical conditions, and is famous for its urban agriculture based on landscape multifunction [41]. The terrain of the study area is saddle-shaped (high at both ends and low in the middle), and its normal landforms include mountains, hills, plains, basins, and downlands. The Qinhuai River runs through the central part of study area from north to south, and the northwest is bordered by the Yangtze River Basin. The moderate subtropical monsoon climate predominates in this area.

### 2.2. Data Collection and Processing

#### 2.2.1. Baidu Heat Map

Baidu Heat Map is a new big data-driven visualization product launched by Baidu in 2014. Based on the geo-location data of mobile phone users on Location Based Services (LBS) platform, the product presents users with varying degrees of population clustering by means of certain spatial representation processing; that is, different color blocks are superimposed on the online map to describe the distribution of people in the city in real time [42].

**Figure 2:**
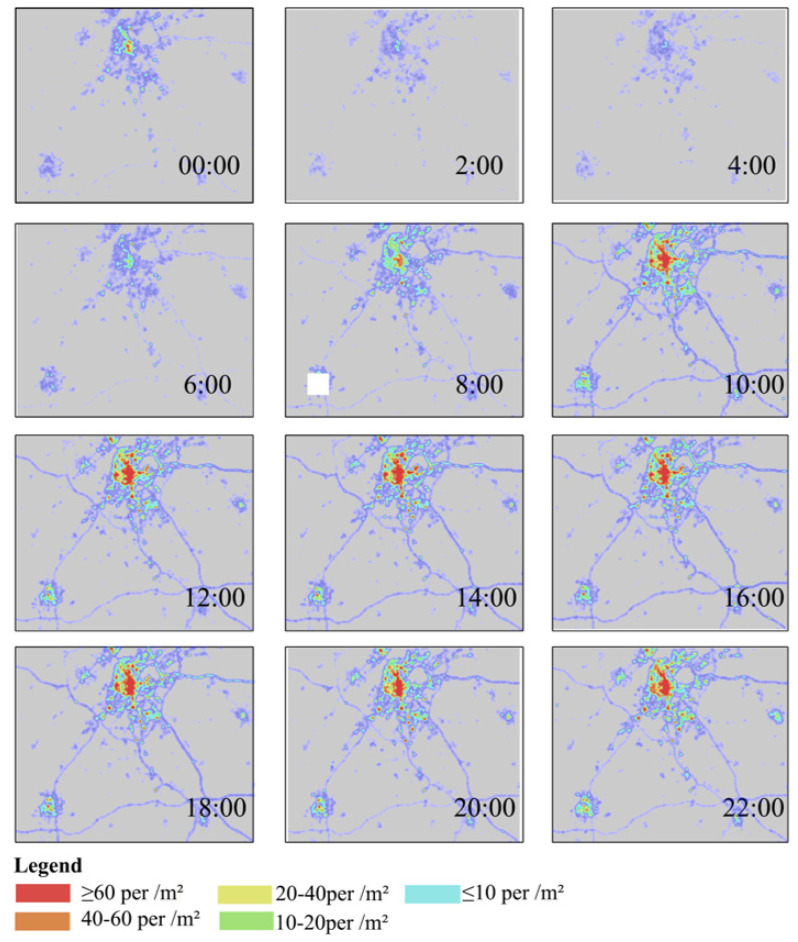
The population of Baidu heat map change over 24 h on September 27 (weekend).

The Baidu Heat Map data in Jiangning District, Nanjing was tracked for 7 days from 27 September to 3 October 2019 (including working days and holidays), and a self-compiled program was used for timed interception of the Heat Map data (24 h a day, at an interval of 2 h); a total of 168 heat maps were captured and used as the important basic data for the study. The study attaches importance to the relative situation of population agglomeration and distribution in different areas within different time periods. For the convenience of data analysis, the heat intensity was used to measure the density reflected by the heat map. The areas in different colors were assigned with heat intensity values; a higher heat intensity indicates a denser population, while a lower intensity indicates a sparser population (Figure 2).

#### 2.2.2. Landscape Functions Data

**Figure 3:**
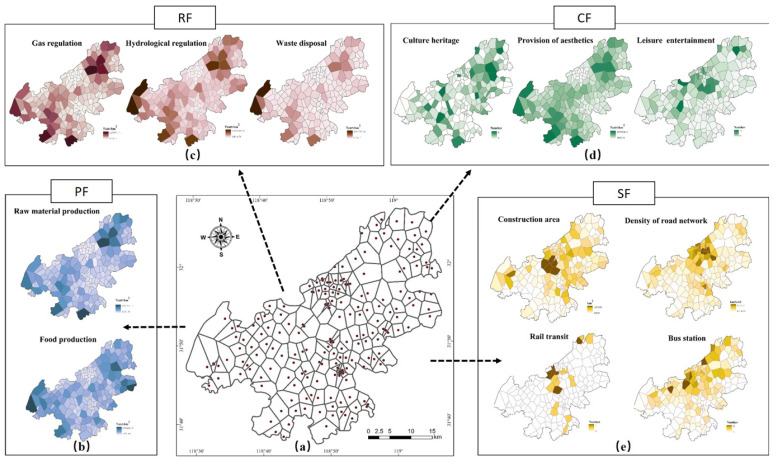
Spatial distribution characteristics of LFs in Jiangning District, Nanjing (**a**) the Tyson Polygon buffer of rural community (**b**) spatial distribution map of regulating function (**c**) spatial distribution map of Culture function (**d**) spatial distribution map of provision function (**e**) spatial distribution map of support function.

The present study divides the LFs into four categories by taking into account the previous studies [43], i.e., provision function (PF), regulation function (RF), culture function (CF), and support function (SF) (Table 1). The provision function means to provide food, forests, water, and other products to humans, and reflects the resources produced by the natural ecosystem; the regulation function means to regulate the climate and improve the environment in suburban rural communities; the tourism resources and location advantages of the suburban rural communities meet the spiritual needs of humans for leisure and recreation, thus being brought under the culture function; the support function incorporates the relevant infrastructures that provide the space or ground for most human activities. The spatial radiation area of each rural community point was obtained based on the Thiessen polygon buffer established with rural community as the center. Then, normalization was performed based on the land use classification map, the Point of Interests (POI) data of recreation and entertainment and other quantitative indicators to achieve the values of LFs (Figure 3). Min-max normalization is used to change the data to a fixed range [0,1] without changing the data distribution. The author used this as study data for further analysis.

### 2.3. Analysis Method

**Figure 4:**
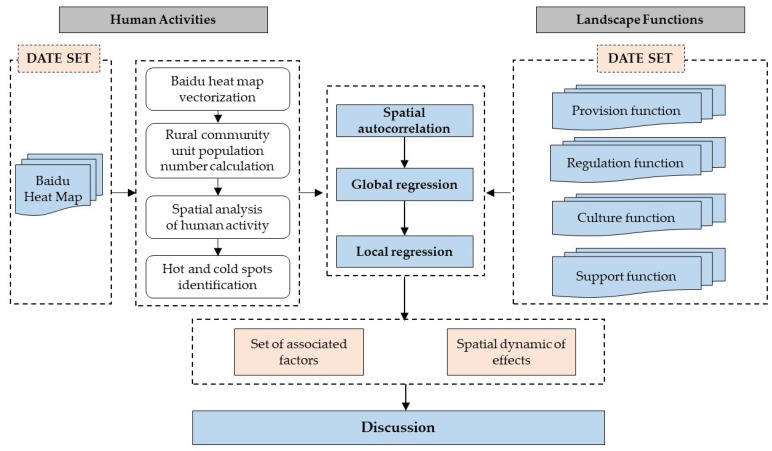
The flowchart of the research process.

The purpose of this study is to take into account the LFs and use the data related to human activities (Baidu heat intensity) to make correlation analysis so as to investigate and describe the impact between LFs and human activities. This study is divided into four parts. First, the obtained Baidu Heat Maps are vectorized in ArcGIS Pro, and then to statistics the average population of different rural community units according to color value; then, the intensity of human activity is subjected to spatial heterogeneity analysis (Moran’s I); next, the ordinary least squares (OLS) model is used for exploratory analysis to screen and evaluate the variables that were not collinear in the LFs factors; finally, the geographically weighted regression (GWR) model is used to analyze the changes in the relationship between human activities and LFs, and then to explore the driving factors affecting human activities; then, such factors are visualized (Figure 4).

#### 2.3.1. Quantified Impact of Human Activities

The data collected are respectively subjected to defined projection and Georeferencing using the ArcGIS pro tool. The data are divided by rural community unit, and then categorized and stored in the unit database. Then, the color value of each community unit is identified, and the statistics of active population of each unit is made based on color values. The intensity of human activity of each unit is the sum of the active population of each pixel unit in the unit, and is characterized by the sum of the products of pixel size and its population:

(1) $$D_{n}=\sum_{i=1}^{h}\delta_{ni}\times p_{i}$$

where *n* represents different areas; $D_{n}$ stands for the Intensity of human activity in the unit; $P_{i}$ denotes the active population represented by class-*i* color value in the area; $\delta_{ni}$ is the total area of class-*i* pixel size in the area; *h* means the total number of categories of color values.

The $n_{i}$ estimated in the paper refers to the official legends of Baidu, while the color value and brightness together represent population: Red means “very crowded” (population density > 60 people/square meter); orange means “crowded” (population density > 40–60 people/square meter); yellow means “moderate” (population density > 20–40 people/square meter); light green means “comfortable” (population density > 10–20 people/square meter); blue and purple indicate “very comfortable” (population density ≤ 10 people/square meter).

#### 2.3.2. Spatial Autocorrelation between Human Activities and Landscape Functions

To assess the existence of the overall spatial autocorrelation between human activities and LF or the signs of spatial heterogeneity, the global Moran’s *I* is used to calculate the global spatial autocorrelation of selected variables of different community units. The global Moran’s *I* is a global measure of spatial autocorrelation used to quantify the degree to which regions are aggregated or uniformly distributed in general [44]. The formula is as follows:

(2) $$I=\frac{n}{\sum_{i=1}^{n}\sum_{j}^{n}W_{ij}}\times \frac{\sum_{i=1}^{n}\sum_{j}^{n}W_{ij}\left(x_{i}-\bar{x}\right)\left(x_{j}-\bar{x}\right)}{\sum_{i=1}^{n}{\left(x_{i}-\bar{x}\right)}^{2}}$$

where $x_{i}$ and $x_{j}$ are values of the selected variables; $W_{ij}$ is the geospatial weight connection matrix of *i* and, which is calculated based on Euclidean distance method; *x* denotes the average intensity of human activity of all community units. The global Moran’s *I* value is generally between −1 and 1. If it is smaller than 0, the spatial unit does not have similar attributes and the distribution is discrete; if it tends to 0, it indicates that the spatial units are randomly distributed; if it is greater than 0, the spatial unit attributes are positively correlated.

Since the global Moran’s *I* cannot capture and analyze the local parts, Getis-Ord Gi* analysis is added for human activities; this method is more suitable for describing and visualizing the spatial distribution of human activities and heterogeneous units [44]. The formula is as follows:

(3) $$G_{I}^{*}\left(d\right)=\frac{\sum_{j=1}^{n}W_{ij}X_{j}-\bar{X}\sum_{j=1}^{n}W_{ij}}{\sqrt[{S}]{\left\{[(\sum_{j=1}^{n}W_{ij}^{2})-{\left(\sum_{j=1}^{n}W_{ij}\right)}^{2}]/\left(n-1\right)\right\}}}$$

where *X_j_* represents the human activity intensity at cell *j*; *x* is the average intensity of human activity; *W_ij_* denotes the scale distance of unit *j*; *n* means the number of units.

Finally, the spatial types of the three heterogeneous units for the heat intensity of human activity are identified: Hot spots (rural communities with high heat intensity of human activities); cold spots (rural communities with low heat intensities of human activity); random areas (rural communities with no spatial autocorrelation).

#### 2.3.3. Screening of LF Factor

OLS is an algorithm extensively used for current studies [45]. In this paper, the correlation coefficients of explanatory variables are evaluated through OLS, to screen out and evaluate the variables without collinearity. The OLS model can be expressed as:

(4) $$y=\beta_{0}+\sum_{k=1}^{p}\beta_{k}x_{k}+\varepsilon \;\;$$

where *y* and $x_{k}$ represents the dependent and independent variables, respectively; $\beta_{0}$ is the intercept of sample *k*; p is the total number of spatial units involved in the analysis; $\beta_{k}$ is the local estimated coefficient of the independent variable $x_{k}$; ε denotes the random error.

The probability value (*p*-value) determines the introduction or deletion of variables; the variable is introduced when the probability value is less than 0.05, and deleted when the probability value is more than 0.05. In the result of stepwise linear regression, the variance inflation factor (VIF) is used to explain the linear correlation between independent variables. VIF is a measure of the severity of complex (multiple) collinearity in multiple linear regression model; if VIF is greater than 10, the multicollinearity is serious, and variables that are collinear with others will be deleted.

The Koenker’s studentized Bruesch–Pagan (Koenker (BP)) statistics are used to determine whether the explanatory variable in the model has a consistent relationship with the dependent variable [46]; The null hypothesis of this test is that the tested model is steady state. For a confidence of 95%, a *p* value less than 0.05 indicates that the model is statistically significant heteroscedasticity and/or unsteady. Regression models with statistical significance instability are usually well suited for GWR analysis.

#### 2.3.4. Geographical Weighted Regression Analysis

OLS can evaluate the coefficients of explanatory variables, but it lacks spatial non-stationarity test. As a local regression model, GWR model is a spatial analysis technique for estimating parameters based on the traditional regression model (OLS), allowing the study process to directly simulate non-stationarity of different spaces; it reflects the degree of impact of different geographical location variables on the area [47]. Its model structure computing formula is as follows:

(5) $$y_{i}=\beta_{0}\left(u_{i}+v_{i}\right)+\sum_{i=1}^{k}\beta_{k}\left(u_{i}+v_{i}\right)x_{ik}+\varepsilon_{i}$$

where $y_{i}$ is explanatory variable; $\left(u_{i}+v_{i}\right)$ represents geographical coordinates of the *i*-th sample unit; $x_{i}$ means the value of the *k*-th independent variable in the *i*-th sample unit; *k* denotes the number of independent variables; *i* stands for the number of sample units; $\varepsilon_{i}$ is a random disturbance term; $\beta_{k}\left(u_{i}+v_{i}\right)$ is the value of continuous function $\beta_{k}\left(u+v\right)$ in *i* sample unit.

## 3. Results

### 3.1. The Spatial Distribution of Human Activities

**Figure 5:**
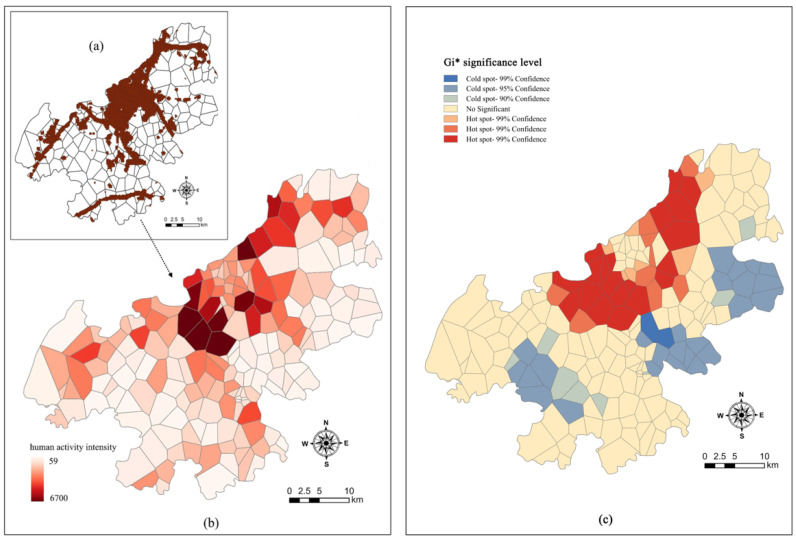
Spatial pattern diagram of human activities in Jiangning District, (**a**) Vectorized Baidu Heat Map, (**b**) Spatial distribution of human activity intensity, (**c**) Spatial distribution map of cold and hot spots of human activities.

The average heat intensity throughout the day is estimated by attaining the vectorized heat map data. As shown in Figure 5, the spatial agglomeration of human activities mainly principally presents a single-core type; with the rural communities of Moling Subdistrict in the central area as the core, the heat intensity diminishes around. All-day high-heat areas are principally observed in the central–northern part of the study area; such areas are substantially close to the main urban area and considered to be inner suburban area. It is worth noting that the communities near the Qinglong Mountain is also a high-heat area for human activities; this area also has a high LF. This demonstrates the fact that there are usually higher human activities under higher LFs; otherwise, there would be fewer human activities.

The heterogeneous units of human activity are identified by Getis-ord Gi*. The Figure 5c shows three heterogeneous units, i.e., hot spot, cold spot, and random area. In space, the heat intensity of human activities in the study area is higher in the north than in the south; there are a lot of hot spots in the north, which correspond to areas with high support functions such as favorable transportation. The cold spots are scattered between the Yangtze River Basin and Fangshan Mountains in the southwest, and in the rural communities far away from the main urban areas in the southwest; most are located in specific areas of land (e.g., arable land). Furthermore, there are two main cold spots of human activities, i.e., the Jingming Village Community and Xinnong Community in Hushu Subdistrict; the significance of both is greater than 0.01.

### 3.2. Spatial Autocorrelation of Human Activities and Landscape Functions

The global Moran’s *I* statistics are used to calculate the spatial autocorrelation of rural variables in Jiangning District. The test in Table 2 shows that the data distribution is only less than 1% likely to be random; the probability of data aggregation is greater than the probability of random distribution; and null hypothesis can be significantly rejected. All explanatory and dependent variables have *p* values that are smaller than 0.01, while the Moran *I* index is between 0 and 1, which indicates a positive spatial correlation pattern.

### 3.3. Explanatory Variable Selection and OLS Analysis Result

First, OLS was used to calculate the overall characteristics of the impact of LF factors on human activities, and select important variables. The result of Model 1 (Table 3) shows that the VIF values of six variables (i.e., waste disposal, hydrological regulation, gas conditioning, raw material production, food production, and provision of aesthetics) are greater than 7.5, which demonstrates that these six variables exhibit obvious multicollinearity. Furthermore, the probability values of rail transit, road network density and cultural heritage are 0.996, 0.060, 0.084, respectively, which are all greater than 0.05; this demonstrates that these three explanatory variables have no significant effect on human activity behavior. However, this result is inconsistent with previous studies; for instance, Jacobs pointed out that there was a significant correlation between spatial vitality and subway stations [48]. Among the other correlated variables, construction area is closely related to human activities, which is consistent with Ye’s study [49]. It is worth noting that the coefficient of cultural heritage is negative, indicating that it has a negative correlation effect on human activities in suburban rural communities. Hence, the following seven variables are retained after the screening of explanatory variables and a number of regressions: Hydrological regulation, provision of aesthetics, construction land, road network density, bus station, cultural heritage, and leisure entertainment (Table 3, Model 2).

Koenker’s (BP) value is 16.125 (*p* = 0.04), which shows that the statistical data are statistically significant and the model is non-stationary. Then, a spatial autocorrelation test is performed on the OLS residuals, indicating a strong autocorrelation. Given the z-score of 4.18, there was less than a one percent likelihood that this clustered pattern could be the result of random chance. These findings imply the influence of complex LFs, so the necessity of local regression is emphasized. The authors retain the factors whose VIF value is less than 10 and the seven factors that have important effect on the independent variables according to the results of stepwise multiple linear regression; then, a GWR model is built to deconstruct the local characteristics of environmental factors’ impact.

### 3.4. The Relationship between Human Activities and Landscape Functions

**Figure 6:**
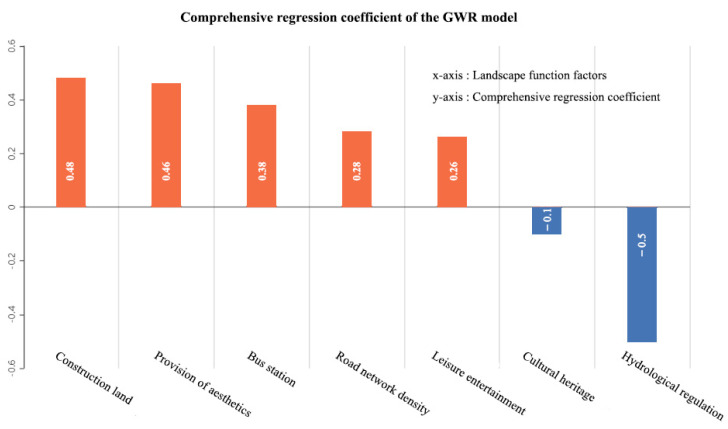
Comprehensive regression coefficient of LF factors of the GWR model.

**Figure 7:**
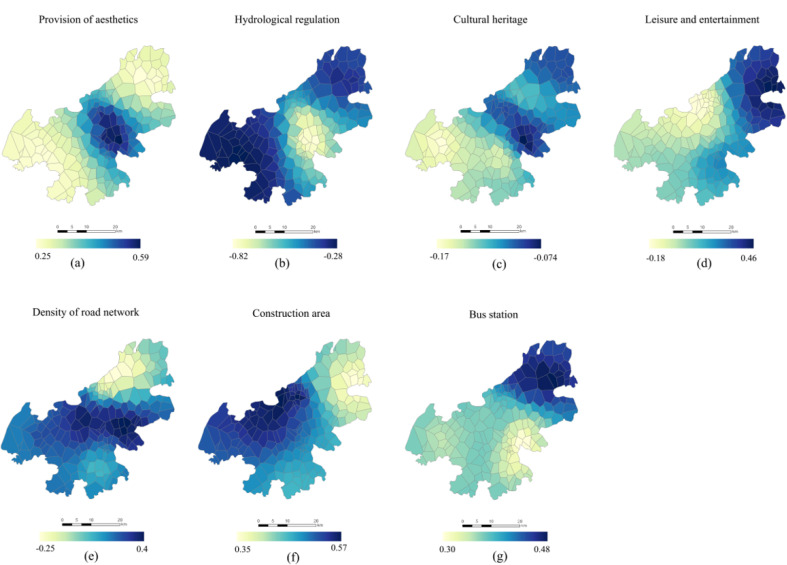
Spatial patterns of regression coefficients between human activities and the factors of LFs, (**a**) Provision of aesthetics, (**b**) Hydrological regulation, (**c**) Cultural heritage, (**d**) Leisure entertainment, (**e**) Road network density, (**f**) Construction area, (**g**) Bus station.

Table 4 shows the final parameters in GWR model. The model goodness of fit reaches 0.809; after the adjustment, the goodness of fit is 0.750, which is higher than that of the OLS model. In addition, the Akechi information criterion (AIC) value drops from 269.45 to 242.40.GWR value is smaller than the OLS value and the difference is more than 3; hence, the information provided by GWR is more specific and reliable than that provided by OLS. The result indicates that the GWR model can better explain the local changes in impact and reduce the spatial autocorrelation of model residuals. The local coefficients between each location and each variable for the 190 rural communities and seven variables are identified through the GWR test. As shown in Figure 6, the effects of variables in the model vary greatly in Jiangning District. A higher coefficient value (positive or negative) indicates that a particular predictor variable has a greater impact on the dependent variable, while a lower coefficient value means a smaller impact. By the comprehensive regression coefficient of each factor of the GWR model, the degrees of their impacts on human activities are in Figure 6:

By visualizing the local coefficients of GWR model for North China, it is observed that LFs have spatial differences in the intensity of human activities. Among the explanatory variables, construction land has the most significant impact on human activities, which demonstrates that socio-economic activities are the main aspect affecting human activities in suburban rural communities. The comprehensive regression coefficient of provision of aesthetics is similar to that of the construction area, but they are significantly different from each other in spatial distribution of impact factor. High values of the impact factors of construction area are principally concentrated in the central and western parts of the study area, while the high values of provision of aesthetics are principally observed in the central part, where the degree of impact diminishes around, exhibiting an obvious spatial differentiation. High values of bus station and leisure are concentrated in the northeast of the study area (around the Qinglong Mountain), which happens to be the low-value area of impact factor of the road network density. The Qinglong Mountain provides a large number of natural landscapes and tourist attractions, but human activities are subject to some restrictions by the mountains and forests. From an overall perspective, the indicators of hydrological regulation and cultural heritage have a negative impact on human activities, which echoes the OLS result. From the perspective of rural community units, Figure 7 shows that hydrological regulation has a greater negative driving effect on human activities in the central part of the study area; such effect diminishes from the center to the periphery. This indicates that the intensity of human activities decreases with the increase of hydrological function. Moreover, cultural heritage has a certain negative impact in the west (around the Yangtze River), and has a positive driving effect on the rest of the area.

## 4. Discussion

### 4.1. Relationship between Landscape Functions and Human Activities

Human activities have long been the focus of studies on suburban rural communities, since they are closely related to the dynamic changes of rural space. However, research findings of big data are mostly focused on urban space, and they are still rare when it comes to the community scale. As the fundamental task of China’s rural revitalization, community-scale evaluation of human activities helps to reveal the changes in human demand for the LF in suburban rural communities in the context of the current urban and rural spatial reconstruction. The importance of some landscape configurations should be considered when improving and maintaining ecosystem services and human well-being; however, how to identify and design is the core issue of sustainable design [50]. Compared with ecosystem service, LFs and landscape characteristics are more closely related to human activities. Therefore, by identifying the key drivers that directly or indirectly affect human activities, it is possible to learn the complex relationship between humans and landscape environment which may be a reasonable way of sustainable landscape development [51].

Taking into account the different characteristics of OLS and GWR models, the authors investigate the impact of human activities and LFs based on the OLS and GWR models. The regulation and provision functions of landscape have a significant negative relationship with the intensity of human activities. In other words, the intensity of human activities is low where the regulation and pro-vision functions are high. For example, hydrologic regulation in the regulation function is negatively correlated with the intensity of human activities, because the main provider of hydrologic regulation is water area (functional value accounted for 47.58% [52]), but the water area that can provide human activities is limited, so the intensity of human activities is relatively low. On the contrary, human activities are bound to change the land habitat and ecological structure, which will have a certain negative impact on the ecosystem. Previous studies have already verified this view [53,54]. An unexpected result is that there is no significant relationship between subway stations and human activities, which is different from other related research findings [47]. That is, because there are fewer subway stations in suburban rural communities, and such stations lead to limited areas; as a result, people travel mostly by buses, taxis, and private cars. This deserves the attention of decision makers.

**Figure 8:**
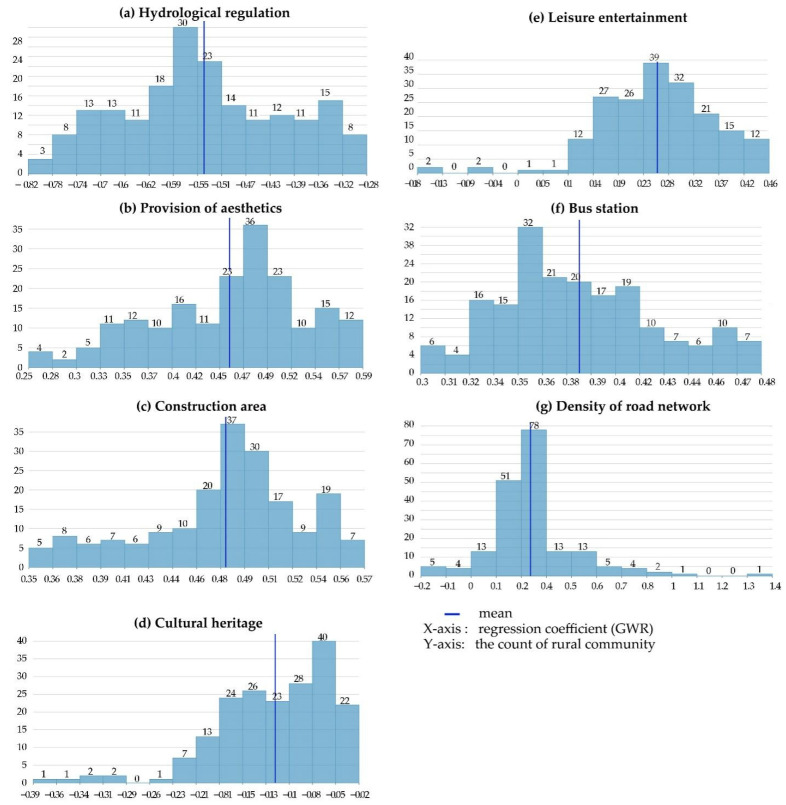
The histogram of regression coefficients between human activities and the factors of LFs, (**a**) The histogram of hydrological regulation, (**b**) The histogram of provision of aesthetics, (**c**) The histogram of construction land, (**d**) The histogram of cultural heritage, (**e**) The histogram of leisure entertainment, (**f**) The histogram of bus station, (**g**) The histogram of density of road network.

According to GWR results and Figure 8, construction area is the main driving force for increasing human activities. In addition, 62.6% of the 190 rural communities (119) had higher impact coefficients than the average level, indicating that the level of land use in Jiangning District was high; previous studies have shown that the land use levels may affect human activities [55]. At the factor level, more urban facilities attract more people. Coincidentally, the area that provided an above-average impact coefficient for aesthetics was the same as the built-up area (62.6%). The reason may be that the main provider of aesthetics are grassland and construction land, so it also has a strong driving effect on human activities. Bus stations, road network density, and leisure entertainments exhibit positive impacts. However, the difference is that the number of rural communities affected by bus stops is more equal. Study reported that the coverage of bus stations is significantly related with population density while the density of bus stations is significantly related with the urban GDP per capita. In China, more than 92% of human activities are found within the 500 m bus service coverage [56], which is to say, the layout of the bus stations meets the needs of most people for activities and facilities. However, the influence coefficients of road network density and leisure and recreation are polarized. The rural communities which are less affected by road network density and more affected by leisure and recreation account for more than 60%. Among them, there are four rural communities where leisure entertainment has a negative impact on human activities. These rural communities are located in the outer suburbs, the furthest distance from the main urban area. By contrast, the negative impacts of cultural heritage and hydrological regulation on human activities exhibit obvious spatial heterogeneity. There are 103 rural communities with lower than average negative impacts of cultural heritage on human activities, all of which are concentrated around the Qinglong Mountains and the central region. Convenient geographical transportation will enhance the attractiveness of cultural heritage, thereby increasing the frequency of human activities [57]. The areas that provide these functions are usually relatively inaccessible land (e.g., forest land, arable land, mountainous land, etc.), which limits human activities to a certain extent. Considering spatial heterogeneity more clearly reflects the regional differences of influencing factors, having provided a new perspective for studying the relationship between LFs and human activities in suburban rural communities.

### 4.2. Guidance on Landscape Planning in Suburban Rural Communities of Big Cities

The spatial conditions of suburban rural communities are very different from those of urban areas and peripheral rural areas. What should suburban rural communities provide for urban and rural residents and land use participants? How should we coordinate human activities and LFs? Our task is to find how to use the functional characteristics of LF in suburban rural communities to allocate human activities without impairing the sustainable development of the landscape environment [33]. Many studies have attempted to directly conduct zoning management through the evaluation of LFs [58,59], but they failed to consider the differences between human activities and heterogeneous units, especially at the rural community scale. The results of this study suggest that the regulation function and provision function are negatively related to the human activities in suburban rural communities, while culture function and support function (except cultural heritage and rail transit) have obvious positive impacts on human activities.

Considering the relationship and impact between LFs and human activities, we can provide more reasonable suggestions for the development of suburban rural communities. First, since ecological security is the foundation for development of the suburban rural communities, planners should create different levels of protected areas based on natural succession and human intervention. However, this does not mean blocking access [60]. Instead, reasonable routes can be created for human activities and the commercial sprawl can be restricted. Second, in view of the comparative advantages of different rural communities, villages are encouraged to focus on the development of dominant functions rather than all functions [61]. The homogenization of landscapes in suburban rural communities may have a negative impact on the landscape service system. For instance, rural communities with leisure entertainments and cultural heritage are encouraged to focus on the development of culture functions and strengthen the support function to assist the development of rural tourism. It is important that the culture function has attracted attention and been incorporated into the sustainable landscape management [62]. Where neighboring rural communities have similar LFs, attempts could be made to form rural communities into an area for development. On the one hand, this stimulates the area-wide rural tourism and improves the financial resources of rural communities [63]; on the other hand, the flow of tourists reduces the pressure of population gathering on limited natural resources, helping balance the ecological environment. By doing so, it is possible to achieve both ecological and economic goals based on the development planning for suburban rural communities.

### 4.3. Limitation

The authors’ study employs the effective framework of big data and LF, where multiple regression and OLS are used for further analysis. However, this study still involves limitations and uncertainties. Firstly, when it comes to selection of LFs, the present study principally comprises four representative LFs (12 factors); however, not all the LFs are reflected; for example, no distinction is made between residential land and commercial land under the term “construction land”; studies have shown that they have different effects on human activities: Commercial and public land has positive impact, while residential and industrial land has a negative impact on urban vitality. Secondly, the big data collection from Baidu Heat Map is unstable, because network quality directly affects the integrity of the collected data; moreover, since the resolution of image is not adjustable, there may be problems with uneven spatial coverage and population penetration [22,64], so the quantitative results may involve deviation. A single data source may depict incomplete spatial pattern of human activities [65]. Therefore, to better define human activities, multi-source media social data such as Microblog and heat.qq.com can be used in future. Thirdly, both OLS and GWR models are based on linear regression that deals only with linear interpolation, so both are subject to certain limitations. Accordingly, it is necessary to further optimize the models in future. Finally, the time frame of Baidu thermal map in the study is only 7 days, which may result in incomplete data. The future will extend the time span and deepen the research on the time series of human activities.

## 5. Conclusions

Due to the geographical location and crowd peculiarities, suburban rural communities are normally considered complex and contradictory areas that increasingly demand for LF; meanwhile, LFs varies greatly with the landscape configuration. Hence, it is necessary to study the relationship between LF and human activities in suburban rural communities. Although the interaction between LFs and human activities is extremely strong according to previous studies [39,66], the focus is on how humans affect LF (especially ecological service function) without considering the positive and negative impacts of LFs on human activities. In this study, multi-source data were used to study the correlation and mechanism of human activity intensity and LF in suburban rural communities at regional scale, which can better highlight the characteristics of suburban rural communities. Furthermore, the location and time-based big data offers the ways to track human activities, thereby searching after the spatial patterns of human activities, which leads to this study. First, the Baidu heat intensity was used to quantify human activities. Then, the relationship with human activities was investigated based on the LF, and the principal drivers for human activities were described. The present study demonstrates the following facts: (1) The intensity of human activities presents obvious spatial heterogeneity in the suburban rural communities, and decreases outwards from the central area; the intensity in inner suburban area is significantly higher than in outer suburban area. (2) Construction area, provision of aesthetics, bus station, road network density, and leisure entertainment have obvious positive impacts on human activities in various areas; construction area is the principal driver; cultural heritage, hydrological regulation, and provision of aesthetics are negatively or positively correlated with human activities in various regions. This study provides an analytical framework based on LFs and human activities to understand the relationship between human activities and LFs based on big data, which will help guide the research on suburban and rural landscape planning. In the face of the sustainable development of landscape and the well-being of human beings in suburban rural communities, it is necessary to make reasonable planning and allocation according to the landscape characteristics and human attraction of rural communities, so as to achieve the balanced development of ecological nature and social economy in suburban rural communities.

## Figures and Tables

**Table 1:** The description of landscape function.

Level-I Type	Level-II Type	Description
Provision function (PF)	Food production	Converting solar energy into edible plant and animal products
	Raw material production	Converting solar energy into biological energy for building or other purposes
Regulating function (RF)	Gas regulation	The ecosystem maintains the balance of atmospheric chemical components, absorbing SO_2_, fluorides, and nitrogen oxides
	Hydrological regulation	The freshwater filtration, retention, and storage by the ecosystem, as well as the supply of freshwater
	Waste disposal	The role of vegetation and organisms in the removal and decomposition of excess nutrients and compounds; dust trapped
Culture function (CF)	Provision of aesthetics	Landscapes (potentially) available for entertainment, and valuable in terms of culture and arts
	Cultural heritage	Immovable cultural relics of great historical, artistic, and scientific value.
	Leisure entertainment	Tourist spots offering leisure and entertainment
Support function (SF)	Transport function	Accessibility mapping(density of road network, bus station, and rail transit)
	Construction function	The ability to provide humans with living and working spaces

**Table 2:** Moran’s *I* test of human activities and LFs.

**Variable**	**Moran *I***	***p***	**z**	**Variable**	**Moran *I***	***p***	**z**
Human activities	0.47	0.00	9.83	Hydrological regulation	0.32	0.00	7.04
Road network density	0.67	0.00	13.96	Gas regulation	0.42	0.00	8.79
Bus station	0.41	0.00	8.56	Raw material production	0.39	0.00	8.23
Rail transit	0.33	0.00	7.03	Food production	0.51	0.00	10.54
Cultural heritage	0.13	0.00	2.84	Provision of aesthetics	0.42	0.00	8.80
Leisure entertainment	0.21	0.00	4.53	Construction area	0.31	0.00	6.45
Waste disposal	0.24	0.00	5.56				

**Table 3:** Results of the linear regression analysis. * *p* < 0.05.

	Explanatory Variables	Coefficient	StdError	t-Value	*p*-Value	VIF
**Model 1**	Waste disposal	−10.993	2.306	−4.766	0.000 *	848.645
	Hydrological regulation	−12.110	2.591	−4.674	0.000 *	>1000
	Gas regulation	−2.607	0.944	−2.763	0.000 *	396.959
	Raw material production	−0.383	0.505	−0.758	0.045	90.674
	Food production	−1.300	0.263	−4.934	0.000 *	26.989
	Provision of aesthetics	1.228	0.377	3.252	0.000 *	70.791
	Construction land	0.572	0.074	7.716	0.000 *	2.226
	Road network density	0.100	0.064	1.572	0.060	2.610
	Bus station	0.336	0.069	4.890	0.000 *	2.328
	Rail transit	0.000	0.043	0.008	0.996	1.340
	Cultural heritage	−0.102	0.053	−1.931	0.084	1.227
	Leisure entertainment	0.308	0.063	4.877	0.000 *	1.250
	R ^2^	0.732				
	Adjust R ^2^	0.712				
	F	40.103				
	Sig.	0.000				
**Model 2**	Hydrological regulation	−0.365	0.152	−2.405	0.017 *	4.707
	Provision of aesthetics	0.341	0.109	3.109	0.002 *	5.344
	Construction area	0.450	0.071	6.367	0.000 *	1.807
	Road network density	0.226	0.600	3.787	0.000 *	2.060
	Bus station	0.394	0.071	5.524	0.000 *	2.240
	Cultural heritage	−0.115	0.553	−2.092	0.037 *	1.203
	Leisure entertainment	0.299	0.064	4.652	0.000 *	1.163
	R ^2^	0.692				
	Adjust R ^2^	0.678				
	F	50.836				
	Sig.	0.000				
	AIC	269.45				
	Koenker (BP)	16.125181 * (*p* = 0.040)				

**Table 4:** Calculation results of geographical weighted regression (GWR) model.

R2	AdjR2	AICc	Sigma-Squared MLE
0.806	0.750	242.4	0.009

## Data Availability

The data presented in this study are available on request from the author. The data are not publicly available due to privacy. Images employed for the study will be available online for readers.
